# Optimal clustering with missing values

**DOI:** 10.1186/s12859-019-2832-3

**Published:** 2019-06-20

**Authors:** Shahin Boluki, Siamak Zamani Dadaneh, Xiaoning Qian, Edward R. Dougherty

**Affiliations:** 1 Department of Electrical and Computer Engineering, Texas A&M University, MS3128 TAMU, College Station, 77843 TX USA; 2 TEES-AgriLife Center for Bioinformatics & Genomic Systems Engineering, College Station, 77843 TX USA

**Keywords:** Clustering, Missing data, Optimal design, Pattern recognition

## Abstract

**Background:**

Missing values frequently arise in modern biomedical studies due to various reasons, including missing tests or complex profiling technologies for different omics measurements. Missing values can complicate the application of clustering algorithms, whose goals are to group points based on some similarity criterion. A common practice for dealing with missing values in the context of clustering is to first impute the missing values, and then apply the clustering algorithm on the completed data.

**Results:**

We consider missing values in the context of optimal clustering, which finds an optimal clustering operator with reference to an underlying random labeled point process (RLPP). We show how the missing-value problem fits neatly into the overall framework of optimal clustering by incorporating the missing value mechanism into the random labeled point process and then marginalizing out the missing-value process. In particular, we demonstrate the proposed framework for the Gaussian model with arbitrary covariance structures. Comprehensive experimental studies on both synthetic and real-world RNA-seq data show the superior performance of the proposed optimal clustering with missing values when compared to various clustering approaches.

**Conclusion:**

Optimal clustering with missing values obviates the need for imputation-based pre-processing of the data, while at the same time possessing smaller clustering errors.

**Electronic supplementary material:**

The online version of this article (10.1186/s12859-019-2832-3) contains supplementary material, which is available to authorized users.

## Background

Clustering has been a mainstay of genomics since the early days of gene-expression microarrays [1]. For instance, expression profiles can be taken over various tissue samples and then clustered according to the expression levels for each sample, the aim being to discriminate pathologies based on their differential patterns of gene expression [2]. In particular, model-based clustering, which assumes that the data are generated by a finite mixture of underlying probability distributions, has gained popularity over heuristic clustering algorithms, for which there is no concrete way of determining the number of clusters or the best clustering method [3]. Model-based clustering methods [4] provide more robust criteria for selecting the appropriate number of clusters. For example, in a Bayesian framework, utilizing Bayes Factor can incorporate both *a priori* knowledge of different models, and goodness of fit of the parametric model to the observed data. Moreover, nonparametric models such as Dirichlet-process mixture models [5] provide a more flexible approach for clustering, by automatically learning the number of components. In small-sample settings, model-based approaches that incorporate model uncertainty have proved successful in designing robust operators [6–9], and in objective-based experiment design to expedite the discovery of such operators [10–12].

Whereas classification theory is grounded in feature-label distributions with the error being the probability that the classifier mislabels a point [6, 13]; clustering algorithms operate on random labeled point processes (RLPPs) with error being the probability that a point will be placed into the wrong cluster (partition) [14]. An optimal (Bayes) clusterer minimizes the clustering error and can be found with respect to an appropriate representation of the cluster error [15].

A common problem in clustering is the existence of missing values. These are ubiquitous with high-throughput sequencing technologies, such as microarrays [16] and RNA sequencing (RNA-seq) [17]. For instance, with microarrays, missing data can occur due to poor resolution, image corruption, or dust or scratches on the slide [18], while for RNA-seq, the sequencing machine may fail to detect genes with low expression levels owing to the random sampling nature of sequencing technologies. As a result of these missing data mechanisms, gene expression data from microarray or RNA-seq experiments are usually in the form of large matrices, with rows and columns corresponding to genes and experimental conditions or different subjects, respectively, with some values missing. Imputation methods, such as *MICE* [19], *Amelia II* [20] and *missForest* [21], are usually employed to complete the data matrix before clustering analysis; however, in small-sample settings, which are common in genomic applications, these methods face difficulties, including co-linearity due to potential high correlation between genes in samples, which precludes the successful imputation of missing values.

In this paper we follow a different direction by incorporating the generation of missing values with the original generating random labeled point process, thereby producing a new RLPP that generates the actual observed points with missing values. The optimal clusterer in the context of missing values is obtained by marginalizing out the missing features in the new RLPP. One potential challenge arising here is that in the case of missing values with general patterns, conducting the marginalization can be computationally intractable, and hence resorting to approximation methods such as Monte Carlo integration is necessary.

Although the proposed framework for optimal clustering can incorporate the probabilistic modeling of arbitrary types of missing data mechanisms, to facilitate analysis, throughout this work we assume data are missing completely at random (MCAR) [22]. In this scenario, the parameters of the missingness mechanism are independent of other model parameters and therefore vanish after the expectation operation in the calculation of the posterior of label functions for clustering assignment.

We derive the optimal clusterer for different scenarios in which features are distributed according to multivariate Gaussian distributions. The performance of this clusterer is compared to various methods, including *k*-POD [23] and fuzzy *c*-means with optimal completion strategy [24], which are methods for directly clustering data with missing values, and also *k*-means [25], fuzzy *c*-means [26] and hierarchical clustering [27] with the missing values imputed. Comprehensive simulations based on synthetic data show the superior performance of the proposed framework for clustering with missing values over a range of simulation setups. Moreover, evaluations based on RNA-seq data further verify the superior performance of the proposed method in a real-world application with missing data.

## Methods

### Optimal clustering

Given a point set $S \subset \mathbb {R}^{d}$, where *d* is the dimension of the space, denote the number of points in *S* by *η*(*S*). A *random labeled point process* (RLPP) is a pair (*Ξ*,*Λ*), where *Ξ* is a point process generating *S* and *Λ* generates random labels on point set *S*. *Ξ* maps from a probability space to $[N;\mathcal {N}]$, where *N* is the family of finite sequences in $\mathbb {R}^{d}$ and $\mathcal {N}$ is the smallest *σ*-algebra on *N* such that for any Borel set *B* in $\mathbb { R}^{d}$, the mapping *S*→*η*(*S*∩*B*) is measurable. A random labeling is a family, *Λ*={*Φ*_*S*_:*S*∈*N*}, where *Φ*_*S*_ is a random label function on the point set *S* in *N*. Denoting the set of labels by *L*={1,2,...,*l*}, *Φ*_*S*_ has a probability mass function on *L*^*S*^ defined by *P*_*S*_(*ϕ*_*S*_)=*P*(*Φ*_*S*_=*ϕ*_*S*_|*Ξ*=*S*), where *ϕ*_*S*_:*S*→*L* is a deterministic function assigning a label to each point in *S*.

A label operator *λ* maps point sets to label functions, *λ*(*S*)=*ϕ*_*S*,*λ*_∈*L*^*S*^. For any set *S*, label function *ϕ*_*S*_ and label operator *λ*, the *label mismatch error* is defined as

1 $$ \epsilon_{\lambda }(S,\phi_{S})=\frac{1}{\eta (S)}\sum_{x\in S}I_{\phi_{S}(x)\neq \phi_{S,\lambda }(x)},  $$

where *I*_*A*_ is an indicator function equal to 1 if *A* is true and 0 otherwise. The *error of label function* *λ*(*S*) is computed as $ \epsilon _{\lambda }(S)=\mathbb {E}_{\Phi _{S}}[\epsilon _{\lambda }(S,\phi _{S})|S]$, and the *error of label operator* *λ* for the corresponding RLPP is then defined by $\epsilon \lbrack \lambda ]=\mathbb {E}_{\Xi }\mathbb {E}_{\Phi _{\Xi }}[\epsilon _{\lambda }(\Xi,\phi _{\Xi })]$.

Clustering involves identifying partitions of a point set rather than the actual labeling, where a partition of *S* into *l* clusters has the form $ \mathcal {P}_{S} = \{S_{1},S_{2},...,S_{l} \}$ such that *S*_*i*_’s are disjoint and $ S = \bigcup _{i=1}^{l} S_{i}$. A cluster operator *ζ* maps point sets to partitions, $\zeta (S)=\mathcal {P}_{S,\zeta }$. Considering the label switching property of clustering operators, let us define *F*_*ζ*_ as the family of label operators that all induce the same partitions as the clustering operator *ζ*. More precisely, a label function *ϕ*_*S*_ induces partition $\mathcal {P}_{S} = \{S_{1},S_{2},...,S_{l} \}$, if *S*_*i*_={*x*∈*S*:*ϕ*_*S*_(*x*)=*l*_*i*_} for distinct *l*_*i*_∈*L*. Thereby, *λ*∈*F*_*ζ*_ if and only if *ϕ*_*S*,*λ*_ induces the same partition as *ζ*(*S*) for all *S*∈*N*. For any set *S*, label function *ϕ*_*S*_ and cluster operator *ζ*, define the *cluster mismatch error* by

2 $$ \epsilon_{\zeta}(S,\phi_{S}) = {\underset{\lambda \in F_{\zeta}}{\min}} \epsilon_{\lambda}(S,\phi_{S}),  $$

the *error of partition* *ζ*(*S*) by $\epsilon _{\zeta }(S) = \mathbb {E}_{\Phi _{S}} [\epsilon _{\zeta }(S,\phi _{S})|S ]$ and the *error of cluster operator* *ζ* for the RLPP by $\epsilon [\zeta ] = \mathbb {E}_{\Xi } \mathbb {E}_{\Phi _{\Xi }} [\epsilon _{\zeta }(\Xi,\phi _{\Xi })]$.

As shown in [15], error definitions for partitions can be represented in terms of risk with intuitive cost functions. Specifically, define $G_{\mathcal {P}_{S}}$ such that $\phi _{S} \in G_{\mathcal {P}_{S}}$ if and only if *ϕ*_*S*_ induces $\mathcal {P}_{S}$. The error of partition can be expressed as

3 $$ \epsilon_{\zeta}(S) = \sum_{\mathcal{P}_{S} \in \mathcal{K}_{S}} c_{S}(\zeta(S), \mathcal{P}_{S}) P_{S}(\mathcal{P}_{S}),  $$

where $\mathcal {K}_{S}$ is the set of all possible partitions of *S*, $P_{S}(\mathcal {P}_{S}) = \sum _{\phi _{S} \in G_{\mathcal {P}_{S}}} P_{S}(\phi _{S})$ is the probability mass function on partitions $\mathcal {P}_{S}$ of *S*, and the *partition cost function* between partitions $\mathcal {P}_{S}$ and $\mathcal {Q}_{S}$ of *S* is defined as

4 $$ c_{S}(\mathcal{Q}_{S},\mathcal{P}_{S})=\frac{1}{\eta (S)} {\underset{\phi_{S, \mathcal{Q}_{S}}\in G_{\mathcal{Q}_{S}}}{\min}}\sum_{x\in S}I_{\phi_{S,\mathcal{P} _{S}}\neq \phi_{S,\mathcal{Q}_{S}}},  $$

with $\phi _{S,\mathcal {P}_{S}}$ being any member of $G_{\mathcal {P}_{S}}$. A Bayes cluster operator *ζ*^∗^ is a clusterer with the minimal error *ε*[*ζ*^∗^], called the *Bayes error*, obtained by a Bayes partition, *ζ*^∗^(*S*) for each set *S*∈*N*:

5 $$\begin{array}{@{}rcl@{}} \zeta^{*}(S) &=& \arg {\underset{\zeta(S) \in \mathcal{K}_{S}}{\min}} \epsilon_{\zeta}(S)  \\ &=& \arg {\underset{\zeta(S) \in \mathcal{K}_{S}}{\min}} \sum_{\mathcal{P}_{S} \in \mathcal{K }_{S}} c_{S}(\zeta(S),\mathcal{P}_{S}) P_{S}(\mathcal{P}_{S}).  \\ \end{array} $$

The Bayes clusterer can be solved for each fixed *S* individually. More specifically, the search space in the minimization and the set of partitions with known probabilities in the summation can be constrained to subsets of $\mathcal {K}_{S}$, denoted by $\mathcal {C}_{S}$ and $\mathcal {R}_{S}$, respectively. We refer to $\mathcal {C}_{S}$ and $\mathcal {R}_{S}$ as the set of candidate partitions and the set of reference partitions, respectively. Following [15], we can search for the optimal clusterer based on both optimal and suboptimal procedures (detailed in “Results and discussion” section) with derived bounds that can be used to optimally reduce the size of $\mathcal {C}_{S}$ and $\mathcal {R}_{S}$.

### Gaussian model with missing values

We consider an RLPP model that generates the points in the set *S* according to a Gaussian model, where features of *x*∈*S* can be missing completely at random due to a missing data mechanism independent of the RLPP. More precisely, the points *x*∈*S* with label *ϕ*_*S*_(*x*)=*i* are drawn independently from a Gaussian distribution with parameters *ρ*_*i*_={*μ*_*i*_,*Σ*_*i*_}. Assuming *n*_*i*_ sample points with label *i*, we divide the observations into *G*_*i*_≤*n*_*i*_ groups, where all *n*_*ig*_ points in group *g* have the same set, *J*_*ig*_, of observed features with cardinality |*J*_*ig*_|=*d*_*ig*_. Denoting by *S*_*ig*_ the set of sample points in group *g* of label *i*, we represent the pattern of missing data in this group using a *d*_*ig*_×*d* matrix *M*_*ig*_, where each row is a *d*-dimensional vector with a single non-zero element with value 1 corresponding to the observed feature’s index. Thus, the non-missing portion of sample point *x*∈*S*_*ig*_, i.e. *M*_*ig*_*x*, has Gaussian distribution $ \mathrm {N}(M_{ig}\mu _{i},M_{ig}\Sigma _{i} M_{ig}^{T})$.

Given *ρ*={*ρ*_1_,*ρ*_2_,...,*ρ*_*l*_} of independent parameters, to evaluate the posterior probability of random labeling function *ϕ*_*S*_∈*L*^*S*^, we have

6 $$\begin{array}{*{20}l} P_{S}& (\phi_{S})\propto P(\phi_{S})f(S|\phi_{S})=  \\ & P(\phi_{S})\int f(S|\phi_{S},\rho)f(\rho)d\rho =  \\ & P(\phi_{S})\prod_{\substack{ i=1 \\ n_{i}\geq 1}}^{l}\int \left(\prod_{x\in S_{i}}f_{i}(x|\rho_{i})\right)f(\rho_{i})d\rho_{i}=  \\ & P(\phi_{S})\prod_{\substack{ i=1 \\ n_{i}\geq 1}}^{l}\int \left(\begin{array}{c}\prod_{g=1}^{G_{i}}\prod_{x\in S_{ig}}\end{array}\right. \\ &\left.\begin{array}{c} {N}\left(M_{ig}x;M_{ig}\mu_{i},M_{ig}\Sigma_{i}M_{ig}^{T}\right)\end{array}\right)f(\mu_{i},\Sigma_{i})d\mu_{i}d\Sigma_{i},  \end{array} $$

where *P*(*ϕ*_*S*_) is the prior probability on label functions, which we assume does not depend on the specific points in *S*.

#### Known means and covariances

When mean and covariance parameters of label-conditional distributions are known, the prior probability *f*(*μ*_*i*_,*Σ*_*i*_) in (6) is a point mass at *ρ*_*i*_={*μ*_*i*_,*Σ*_*i*_}. Thus,

7 $$  \begin{aligned} &P_{S}(\phi_{S})\propto P(\phi_{S}) \times \\ &\prod_{\substack{ i=1 \\ n_{i} \geq 1}}^{l} \prod_{g=1}^{G_i} \prod_{x \in S_{ig}} \left[\begin{array}{c} (2\pi)^{-d_{ig}/2} \left|M_{ig}\Sigma_{i} M_{ig}^{T}\right|^{-1/2} \times \end{array}\right.\\ & \left.\begin{array}{c}\!\!\exp \left\{ \,-\, \frac{1}{2} (x-\mu_{i})^{T} M_{ig}^{T} \left(M_{ig}\Sigma_{i} M_{ig}^{T}\right)^{-1} M_{ig} (x-\mu_{i}) \right\} \end{array}\right]. \end{aligned}  $$

We define the group-*g* statistics of label *i* as

8 $$\begin{array}{@{}rcl@{}} m_{ig} &:=&\frac{1}{n_{ig}}\sum_{x \in S_{ig}}M_{ig}x,  \\ \Psi_{ig} &:=&\sum_{x \in S_{ig}}(M_{ig}x-m_{ig})(M_{ig}x-m_{ig})^{T},  \end{array} $$

where *m*_*ig*_ and *Ψ*_*ig*_ are the sample mean and scatter matrix, employing only the observed *n*_*ig*_ data points in group *g* of label *i*. The posterior probability of labeling function (7) then can be expressed as

9 $$  \begin{aligned} &P_{S}(\phi_{S})\propto P(\phi_{S}) \prod_{\substack{ i=1 \\ n_{i} \geq 1}}^l \prod_{g=1}^{G_i} \\ & \left[\begin{array}{c}\left|2\pi \Sigma_{ig}\right|^{-n_{ig}/2}\exp \left\{- \frac{1}{2}\text{tr}\left(\Psi_{ig}(\Sigma_{ig})^{-1}\right)\right\} \times \end{array}\right.\\ & \left.\begin{array}{c}\exp \left\{-\frac{1}{2}n_{ig}(m_{ig}-M_{ig}\mu_{i})^{T}(\Sigma_{ig})^{-1}(m_{ig}-M_{ig}\mu_{i})\right\} \end{array}\right], \end{aligned}  $$

where $\Sigma _{ig}=M_{ig}\Sigma _{i} M_{ig}^{T}$ is the covariance matrix corresponding to group *g* of label *i*.

#### Gaussian means and known covariances

Under this model, data points are generated according to Gaussians whose mean parameters are random and their covariance matrices are fixed. Specifically, for label *i* we have $\mu _{i} \sim \mathrm {N}(m_{i},\frac {1}{\nu _{i}} \Sigma _{i})$, where *ν*_*i*_>0 and *m*_*i*_ is a length *d* real vector. Thus the posterior of label function given the point set *S* can be derived as

10 $$ {\begin{aligned} &P_{S}(\phi_{S}) \propto P(\phi_{S}) \prod_{\substack{ i=1 \\ n_{i} \geq 1}}^{l} \left[ \prod_{g=1}^{G_i} \left[ |2\pi\Sigma_{ig}|^{-n_{ig}/2} \times \right.\right.\\ & \left.\exp \left\{- \frac{1}{2}\text{tr}\left(\Psi_{ig}(\Sigma_{ig})^{-1}\right)\right\}\right] \times (\nu_{i})^{d/2} |2\pi \Sigma_{i}|^{-1/2}\\ &\int \exp \left\{-\frac{1}{2}\sum_{g=1}^{G_i}n_{ig}(m_{ig}-M_{ig}\mu_{i})^{T}(\Sigma_{ig})^{-1} \right.\\ & \left.\left.(m_{ig}-M_{ig}\mu_{i}) - \frac{\nu_i}{2} (\mu_{i}-m_{i})^{T} \Sigma_{i}^{-1} (\mu_{i}-m_{i}) \right\} d\mu_{i} \right]. \end{aligned}}  $$

By completing the square and using the normalization constant of multivariate Gaussian distribution, the integral in this equation can be expressed as

11 $$ \begin{aligned} & \int \exp \left\{-\frac{1}{2}\left[(\mu_{i}-A_{i}^{-1}b_{i})^{T}A_{i}(\mu_{i}-A_{i}^{-1}b_{i})+ \right.\right.\\ & \left.\left. \sum_{g=1}^{G_{i}}n_{ig}m_{ig}^{T}\Sigma_{ig}^{-1}m_{ig}+\nu_{i}m_{i}^{T}\Sigma_{i}^{-1}m_{i}-b_{i}^{T}A_{i}^{-1}b_{i}\right]\right\}\\ & =|A_{i}/(2\pi)|^{-1/2}\exp \left\{-\frac{1}{2}\left[\sum_{g=1}^{G_{i}}n_{ig}m_{ig}^{T}\Sigma_{ig}^{-1}m_{ig}+ \right.\right.\\ & \quad \quad \left.\left.\nu_{i}m_{i}^{T}\Sigma_{i}^{-1}m_{i}-b_{i}^{T}A_{i}^{-1}b_{i}\right]\right\}, \end{aligned}  $$

where

12 $$\begin{array}{*{20}l} A_{i} &=&\sum_{g=1}^{G_{i}}n_{ig}M_{ig}^{T}\Sigma_{ig}^{-1}M_{ig}+ \nu_{i}\Sigma_{i}^{-1}, \end{array} $$

13 $$\begin{array}{*{20}l} b_{i} &=&\sum_{g=1}^{G_{i}}n_{ig}M_{ig}^{T}\Sigma_{ig}^{-1}m_{ig}+ \nu_{i}\Sigma_{i}^{-1}m_{i}. \end{array} $$

#### Gaussian-Inverse-Wishart Means and Covariances

Under this model, data points are generated from Gaussian distributions with random mean and covariance parameters. More precisely, the parameters associated with label *i* are distributed as ${\mu _{i}|\Sigma _{i} \sim \mathrm {N}\left (m_{i},\frac {1 }{\nu _{i}}\Sigma _{i}\right)}$ and *Σ*_*i*_∼IW(*κ*_*i*_,*Ψ*_*i*_), where the covariance has inverse-Wishart distribution

14 $$ {}f(\Sigma_{i}) = \frac{|\Psi_{i}|^{\kappa_{i}/2}}{2^{\kappa_{i} d/2} \Gamma_{d}(\kappa_{i}/2)} |\Sigma_{i}|^{\frac{\kappa_{i}+d+1}{2}} \exp \left(-\frac{1 }{2} \text{tr}\left(\Psi_{i} \Sigma_{i}^{-1}\right) \right).  $$

To compute the posterior probability of labeling function (6), we first marginalize out the mean parameters *μ*_*i*_ in a similar fashion to (10), obtaining

15 $$ \begin{aligned} P_{S}(\phi_{S}) \propto& P(\phi_{S}) \prod_{\substack{ i=1 \\ n_{i} \geq 1}}^l \int \left[ \prod_{g=1}^{G_{i}}|2\pi \Sigma_{ig}|^{-n_{ig}/2}\times \right.\\ &\exp \left\{- \frac{1}{2}\text{tr}\left(\Psi_{ig}(\Sigma_{ig})^{-1}\right)\right\}\times\\ &(\nu_{i})^{d/2} |\Sigma_{i}|^{-1/2} |A_{i}/(2\pi)|^{-1/2}\times \\ &\exp \left\{ -\frac{1}{2} \left[ \sum_{g=1}^{G_i}n_{ig} m_{ig}^T \Sigma_{ig}^{-1} m_{ig} \right.\right.\\ &+\left.\left.\left.\nu_{i} m_{i}^{T} \Sigma_{i}^{-1} m_{i} - b_{i}^{T} A_{i}^{-1} b_{i} \right] \right\} \right] f(\Sigma_{i}) d\Sigma_{i}. \end{aligned}  $$

The integration in the above equation has no closed form solution, thus we resort to Monte Carlo integration for approximating it. Specifically, denoting the term in the brackets in Eq. (15) as *g*(*Σ*_*i*_), we draw *J* samples $\Sigma _{i}^{(j)} \sim \text {IW}(\kappa _{i},\Psi _{i})$, *j*=1,2,...,*J*, and then compute the integral as $\frac {1}{J} \sum _{j=1}^{J} g(\Sigma _{i}^{(j)})$.

## Results and discussion

The performance of the proposed method for optimal clustering with missing values at random is compared with some suboptimal versions, two other methods for clustering data with missing values, and also classical clustering algorithms with imputed missing values. The performance comparison is carried out on synthetic data generated from different Gaussian RLPP models with different missing probability setups, and also on a publicly available dataset of breast cancer generated by TCGA Research Network (https://cancergenome.nih.gov/). In our experiments, the results of the exact optimal solution for the RLPP with missing at random (Optimal) is provided for smaller point sets, i.e. wherever computationally feasible. We have also tested two suboptimal solutions, similar to the ideas in [15], for an RLPP with missing at random. In the first one (Subopt. Pmax), the set of reference partitions ($\mathcal {R}_{S}$) is restricted to a closed ball of a specified radius centered on the partition with the highest probability, where the distance of two partitions is defined as the minimum Hamming distance between labels inducing the partitions. In both Optimal and Pmax, the reference set is further constrained to the partitions that assign the correct number of points to each cluster, but the set of candidate partitions ($\mathcal {C}_{S}$) includes all the possible partitions of *n* points, i.e. 2^*n*−1^. In the second suboptimal solution (Subopt. Pseed), a local search within Hamming distance at 1 is performed starting from five random initial partitions to approximately find the partition with (possibly local) maximum probability. Then the sets of reference and candidate partitions are constrained to the partitions with correct cluster sizes with a specified Hamming distance from the found (local) maximum probability partition. The bounds derived in [15] for reducing the set of candidate and reference partitions are used, where applicable, in Optimal, Pseed, and Pmax.

In all scenarios, *k*-POD and fuzzy *c*-means with optimal completion strategy (FCM-OCS) are directly applied to the data with missing values. In the simulations in [24], where FCM-OCS is introduced, to initialize cluster centers, the authors apply ordinary fuzzy *c*-means to the complete data, i.e. using knowledge of the missing values. To have a fair comparison with other methods, we calculate the initial cluster centers for FCM-OCS by applying fuzzy *c*-means to the subset of points with no missing features for lower missing rates. For higher missing rates we impute the missing values by the mean of the corresponding feature values across all points, and then apply fuzzy *c*-means to all the points to initialize the cluster centers. In order to apply the classical algorithms, the missing values are imputed according to [28], by employing a multivariate Gibbs sampler that iteratively generates samples for missing values and parameters based on the observed data. The classical algorithms included in our experiments include *k*-means (KM), fuzzy *c*-means (FCM), hierarchical clustering with single linkage (Hier. (Si)), and hierarchical clustering with complete linkage (Hier. (Co)). Moreover, completely random clusterer (Random) results are also included for performance comparisons.

### Simulated data

In the simulation analysis, the number of clusters is fixed at 2, and the dimensionality of the RLPPs (number of features) is set to 5. Additional results for 20 features are provided in Additional file 1. Point generation is done based on a Gaussian mixture model (GMM). Three different scenarios for the parameters of the GMM are considered: *i*) Fixed known means and covariances *ii*) Known covariances and unknown means with Gaussian distributions. *iii*) Unknown means and covariances with Gaussian inverse-Wishart distributions. We select the values of the parameters of the point generation process to have an approximate Bayes error of 0.15. The selected values are shown in Table 1. For the point set generation, the number of points from each cluster is fixed *a priori*. The distributions are first drawn from the assumed model, and then the points are generated based on the drawn distributions. A subset of the points’ features is randomly selected to be hidden based on missing at random with different missing probabilities. Four different setups for the number of points are considered in our simulation analysis: 10 points from each cluster (*n*_1_=*n*_2_=10), 12 points from one cluster and 8 points from the other cluster (*n*_1_=12,*n*_2_=8), 35 points from each cluster (*n*_1_=*n*_2_=35), and 42 points from one cluster and 28 points from the other cluster (*n*_1_=42,*n*_2_=28). When having unequal sized clusters, in half of the repetitions *n*_1_ points are generated from the first distribution and *n*_2_ points from the second distribution, and vice-versa in the other half. In each simulation repetition, all clustering methods are applied to the points to generate a vector of labels that induces a two-cluster partition. The predicted label vector by each method is compared with the true label vector of each point in the point set to calculate the error of that method on that point set. In other words, for each method the number of points assigned to a cluster different from their true one are counted (after accounting for the label switching issue) and divided by the total number of points (*n*=*n*_1_+*n*_2_) to calculate the clustering error of that method on the point set. These errors are averaged across all point sets in different repetitions to empirically estimate the clustering error of each method under a model and fixed missing-value probability. In cases with *n*=70, since applying Optimal and Pmax is computationally prohibitive, we only provide the results for Pseed.

**Table 1:** Parameters for the point generation under three models

Model	Mean vectors	Covariance matrices	Distributions’ hyperparameters
Fixed means and covariances	*μ*_1_=0·**1**_*d*_, *μ*_2_=0.445·**1**_*d*_	*Σ*_1_=*Σ*_2_=0.23·*I*_*d*_	—
Gaussian means and fixed covariances	$\mu _{1} \sim \mathrm {N}\left (m_{1},\frac {1}{\nu _{1}}\Sigma _{1}\right)$, $\mu _{2} \sim \mathrm {N}\left (m_{2},\frac {1}{\nu _{2}}\Sigma _{2}\right)$	*Σ*_1_=*Σ*_2_=0.28·*I*_*d*_	*m*_1_=0·**1**_*d*_, *m*_2_=0.45·**1**_*d*_,
			*ν*_1_=30, *ν*_2_=5
Gaussian means and inverse-Wishart covariances	$\mu _{1} \sim \mathrm {N}\left (m_{1},\frac {1}{\nu _{1}}\Sigma _{1}\right)$, $\mu _{2} \sim \mathrm {N}\left (m_{2},\frac {1}{\nu _{2}}\Sigma _{2}\right)$	*Σ*_1_∼IW(*κ*_1_,*Ψ*_1_),*Σ*_2_∼IW(*κ*_2_,*Ψ*_2_)	*m*_1_=0·**1**_*d*_, *m*_2_=0.45·**1**_*d*_,
			*ν*_1_=30, *ν*_2_=5,
			*Ψ*_1_=*Ψ*_2_=20.7·*I*_*d*_,
			*κ*_1_=*κ*_2_=75

N, IW, **1**_*d*_, and *I*_*d*_ denote Gaussian, inverse-Wishart, column vector of all ones with length *d*, and *d*×*d* idendity matrix, respectively

In Additional file 1, the average clustering errors are shown as a function of the Hamming distance threshold used to define the set of reference partitions in Pmax and Pseed, for different simulation scenarios. From the Figures in Additional file 1, we see that in all cases, the performances of Pmax and Pseed are quite insensitive to the set threshold of the Hamming distance for reference partitions. Note that in these types of figures all the other methods’ performances other than Pmax and Pseed are constant in each plot.

**Fig. 1:**
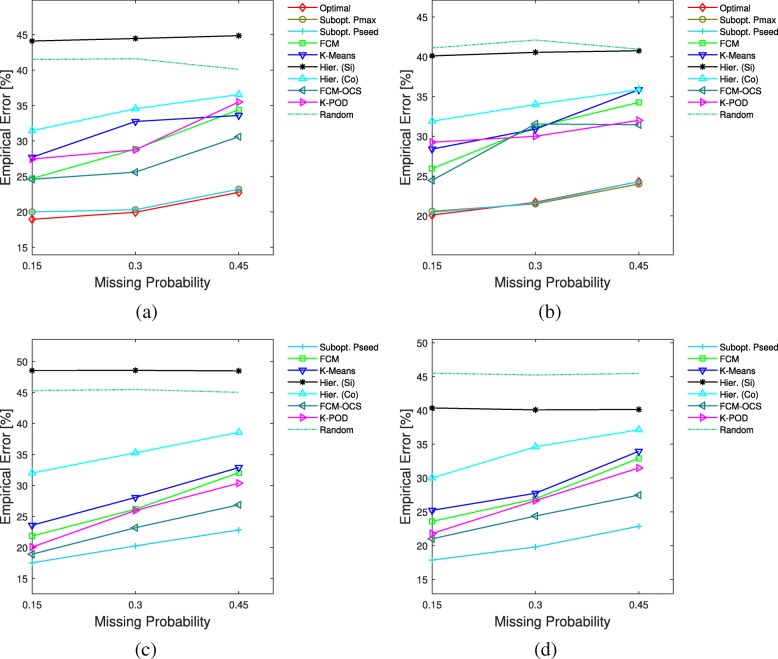
Average clustering errors vs. missing probability for fixed means and covariances model. The first and second rows correspond to *n*=20 and *n*=70, respectively. **a** *n*1=10,*n*2=10, **b** *n*1=12,*n*2=8, **c** *n*1=35,*n*2=35, **d** *n*1=42,*n*2=28

The average results for the fixed mean vectors and covariance matrices across 100 repetitions are shown in Fig. 1. Here, the Hamming distance threshold for reference partitions in Pmax and Pseed is fixed at 1. It can be seen that Optimal, Pmax, and Pseed outperform all the other methods in all the smaller sample size settings, and Pmax and Pseed have virtually the same performance as Optimal. For the larger sample size settings where only Pseed is applied, its superior performance compared with other methods is clear from the figure.

**Fig. 2:**
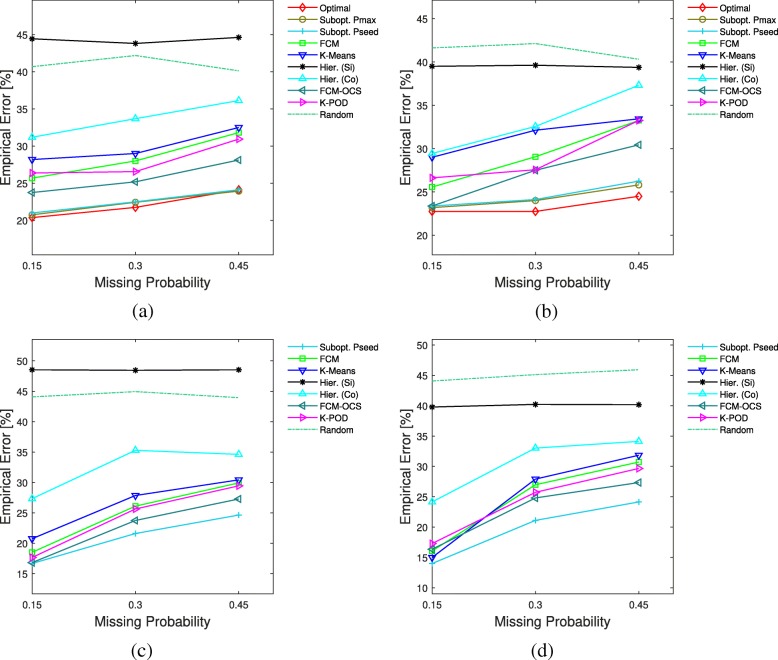
Average clustering errors vs. missing probability for Gaussian means and fixed covariances model. The first and second rows correspond to *n*=20 and *n*=70, respectively. **a** *n*1=10,*n*2=10, **b** *n*1=12,*n*2=8, **c** *n*1=35,*n*2=35, **d** *n*1=42,*n*2=28

Figure 2 depicts the comparison results under the unknown mean vectors with Gaussian distributions and fixed covariance matrices averaged over 80 repetitions. The Hamming distance threshold in Pmax and Pseed is set to 2. For smaller sample sizes, Optimal, Pmax and Pseed have lower average errors than all the other methods. We can see that for balanced clusters the suboptimal and optimal solutions have very close performances, but for the unbalanced clusters case with higher missing probabilities the difference between Optimal and Pmax and Pseed gets noticeable. For larger sample sizes Pseed consistently outperforms the other methods, although for lower missing probabilities it has closer competitors. In all cases, as the missing probability increases, the superior performance of the proposed methods becomes more significant.

**Fig. 3:**
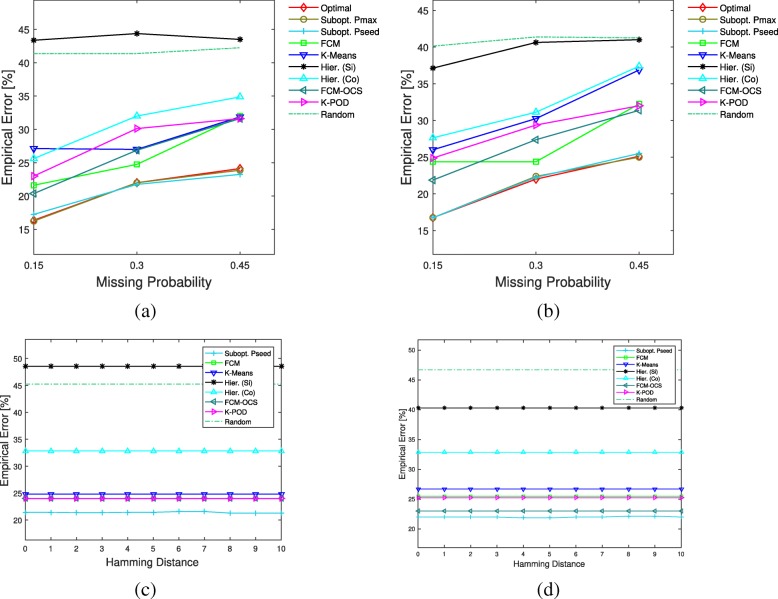
Average clustering errors for Gaussian means and inverse-Wishart covariances model. The first row corresponds to *n*=20, and the errors are shown for different missing probabilities. The second row corresponds to *n*=70 and missing probability of 0.15, where the errors are plotted vs. the Hamming distance threshold used to define the reference partitions in Pseed. **a** *n*1=10,*n*2=10, **b** *n*1=12,*n*2=8, **c** *n*1=35,*n*2=35,miss. prob.=0.15, **d** *n*1=42,*n*2=28,miss. prob.=0.15

The average results under the unknown mean vectors and coavriance matrices with Gaussian-inverse-Wishart distribution over 40 repetitions are provided in Fig. 3. In the smaller sample size cases, the Hamming distance threshold in Pmax and Pseed is fixed at 8, and we can see that the proposed suboptimal (Pmax and Pseed) and optimal clustering with missing values have very close average errors, and all are much lower than the other methods’ errors. For larger sample sizes, only the results for missing probability equal to 0.15 are shown vs. the Hamming distance threshold used to define the reference partitions in Pseed. Again, Pseed performs better than the other methods.

### RNA-seq data

In this section the performance of the clustering methods are examined on a publicly available RNA-seq dataset of breast cancer. The data is available on The Cancer Genome Atlas (TCGA) [29], and is procured by the R package TCGS2STAT [30]. It consists of matched tumor and normal samples, and includes 97 points from each. The original data are in terms of the number of sequence reads mapped to each gene. RNA-seq data are integers, highly skewed and over-dispersed [31–35]. Thus, we apply a variance stabilizing transformation (VST) [36] implemented in DESeq2 package [37], and transform data to a log2 scale that have been normalized with respect to library size. For all subsequent analysis, other than for calculating clustering errors, we assume that the labels of data are unknown. Feature selection is performed in a completely unsupervised manner, since in clustering no labels are known in practice. The top 10 genes in terms of variance to mean ratio of expression are picked as features to be used in clustering algorithms. In general, for setting prior hyperparameters, external sources of information like signaling pathways, where available, can be leveraged [38, 39]. Here, we only use a subset of the discarded gene expressions, i.e. the next 90 top genes (in terms of variance to mean ratio of expression), for prior hyperparameters calibration for the optimal/suboptimal approaches. We follow the approach in [40] and employ the method of moments for prior calibration, but unlike [40], a single set of hyperparameters is estimated and used for both clusters, since the labels of data are not available. It is well known that in small sample size settings, estimation of covariance matrices, scatter matrices and even mean vectors may be problematic. Therefore, similar to [40], we assume the following structure

$$\begin{aligned} & \Psi_{0}=\Psi_{1}= \begin{bmatrix} \sigma^{2} & \rho \sigma^{2} & \dots & \rho \sigma^{2} \\ \rho \sigma^{2} & \sigma^{2} & \dots & \rho \sigma^{2} \\ \vdots & \vdots & \ddots & \vdots \\ \rho \sigma^{2} & \dots & \dots & \sigma^{2} \end{bmatrix} _{d\times d}, \\ & m_{0}=m_{1}=m[1,\cdots,1]_{d}^{T}, \\ & \nu_{0}=\nu_{1}=\nu,\kappa_{0}=\kappa_{1}=\kappa, \end{aligned} $$

and estimate five scalars (*m*, *σ*^2^, *ρ*, *κ*, *ν*) from the data.

**Fig. 4:**
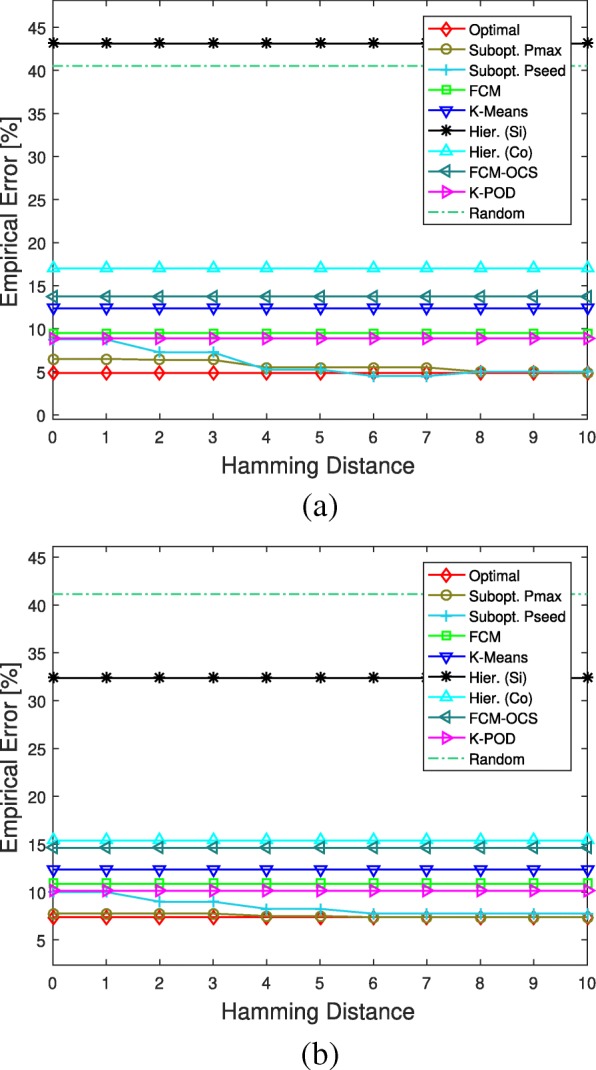
Empirical clustering errors on breast cancer RNA-seq data. **a** *n*1=10,*n*2=10,miss. prob.=0.15, **b** *n*1=12,*n*2=8,miss. prob.=0.15

In each repetition, stratified sampling is done, i.e. *n*_1_ and *n*_2_ points are sampled randomly from each group (normal and tumor). When *n*_1_≠*n*_2_, in half of the repetitions *n*_1_ and *n*_2_ points are randomly selected from the normal and tumor samples, respectively, and vice-versa in the other half. Prior calibration is performed in each repetition, and 15% of the selected features are considered as missing values. Similar to the experiments on the simulated data, the clustering error of each method in each iteration is calculated by comparing the predicted labels and true labels of the sampled points (accounting for label switching issue), and the average results over 40 repetitions are provided in Fig. 4. It can be seen that the proposed optimal clustering with missing values and its suboptimal versions outperform the other algorithms. It is worth noting that the performance of Pseed is more sensitive to the selected Hamming distance threshold for reference partitions compared with the results on simulated data.

## Conclusion

The methodology employed in this paper is very natural. As with any signal processing problem, the basic problem is to find an optimal operator from a class of operators given the underlying random process and a cost function, which is often an error associated with operator performance. While it may not be possible to compute the optimal operator, one can at least employ suboptimal approximations to it while knowing the penalties associated with the approximations.

In this paper, we have, in effect, confronted an old problem in signal processing: If we wish to make a decision based on a noisy observed signal, is it better to filter the observed signal and then determine the optimal decision on the filtered signal, or to find the optimal decision based directly on the observed signal? The answer is the latter. The reason is that the latter approach is fully optimal relative to the actual observation process, whereas, even if in the first approach the filtering is optimal relative to the noise process, the first approach produces a composite of two actions, filter and decision, each of which is only optimal relative to a portion of the actual observation process. In the present situation involving clustering, in the standard imputation-followed-by-clustering approach, it is typically the case that neither the filter (imputation) nor the decision (clustering) is optimal, so that even more advantage is obtained by optimal clustering over the missing-value-adjusted RLPP.

## Additional file

Additional file 1 Supplementary materials. Additional figures are given in a single multi-page PDF. (PDF 516 KB)

## Abbreviations

FCM-OCS: Fuzzy *c*-means with optimal completion strategy
GMM: Gaussian mixture model
Hier. (Co): Hierarchical clustering with complete linkage
Hier. (Si): Hierarchical clustering with single linkage
MCAR: Missing completely at random
RLPP: Random labeled point process
RNA-seq: RNA sequencing
TCGA: The Cancer Genome Atlas
VST: Variance stabilizing transformation
