# Supplementary material for: Optimal clustering with missing values
Source: BMC Bioinformatics. 2019 Jun 20;20(Suppl 12):321. doi: 10.1186/s12859-019-2832-3 (PMC6584727; doi:10.1186/s12859-019-2832-3)
Supplement: Supplementary file 1 — Supplementary materials. Additional figures are given in a single multi-page PDF. (PDF 516 KB) [file 12859_2019_2832_MOESM1_ESM.pdf]

RESEARCH

Supplementary materials for optimal clustering with missing values

Shahin Boluki[1†], Siamak Zamani Dadaneh[1†], Xiaoning Qian[1,2] and Edward R Dougherty[1,2*]

Abstract

This document contains the supplementary figures of the paper "Optimal clustering with missing values".

Additional figures for experiments on simulated data

In all the figures, the average clustering errors are shown in each plot as a function of the Hamming distance threshold used to define the set of reference partitions in Pmax and Pseed. Thus, all the other methods' performances other than Pmax and Pseed are constant in each plot.

Simulations with 20 features

The average clustering errors of different methods across 40 repetitions for the data simulated based on (1) the fixed mean vectors and covariance matrices model, (2) the unknown mean vectors with Gaussian distributions and fixed covariance matrices model, and (3) the unknown mean vectors and coavriance matrices with the Gaussian-inverse-Wishart distribution model are shown in Figures S1, S2, and S3, respectively. We select the values of the parameters of the point generation process to have an approximate Bayes error of 0.15. The selected values are shown in Table S1. In all cases the missing probability is set to 0.2. From the figures we can see that our proposed methods (Optimal, Pmax, and Pseed) outperform all the other methods in all the simulation scenarios with 20 features.

Simulations with 5 features

The average clustering errors of different methods are computed for the data simulated based on the models with (1) the fixed mean vectors and covariance matrices across 100 repetitions, (2) the unknown mean vectors with Gaussian distributions and fixed covariance matrices across 80 repetitions, and (3) the unknown mean vectors and coavriance matrices with the Gaussian-inverse-Wishart distribution setup across 40 repetitions. They are shown in Figures S4, S5, and S6, respectively.

For the Gaussian-inverse-Wishart model (cases with unknown mean and covariances), when having larger sample sizes, only the results for missing probability equal to 0.15 are provided (in the main text), due to the combined complexity of point process based clustering and partition probability computations that increases with the number of missing values.

We can see that in all cases, the performance of Pmax and Pseed is not very sensitive to the set threshold of Hamming distance for reference partitions. For cases with larger n, Pseed's performance is virtually constant as a function of the set Hamming distance.

*Correspondence: edward@ece.tamu.edu
[1]Department of Electrical and Computer Engineering, Texas A&M University, MS3128 TAMU, 77843, College Station, TX, USA
[2]TEES-AgriLife Center for Bioinformatics & Genomic Systems Engineering, 77843, College Station, TX, USA
Full list of author information is available at the end of the article
†Equal contributor

Table S1: Parameters for the point generation under three models for 20 features ($d = 20$). N, IW, $\mathbf{1}_d$, and I_d denote Gaussian, inverse-Wishart, column vector of all ones with length d, and $d \times d$ idendity matrix, respectively.

Model	Mean vectors	Covariance matrices	Distributions' hyperparameters
Fixed means and covariances	$\mu_1 = 0 \cdot \mathbf{1}_d$, $\mu_2 = 0.22 \cdot \mathbf{1}_d$	$\Sigma_1 = \Sigma_2 = 0.23 \cdot I_d$	—
Gaussian means and fixed covariances	$\mu_1 \sim \mathsf{N}(m_1, \frac{1}{\nu_1}\Sigma_1)$, $\mu_2 \sim \mathsf{N}(m_2, \frac{1}{\nu_2}\Sigma_2)$	$\Sigma_1 = \Sigma_2 = 0.28 \cdot I_d$	$m_1 = 0 \cdot \mathbf{1}_d$, $m_2 = 0.22 \cdot \mathbf{1}_d$, $\nu_1 = 120$, $\nu_2 = 20$
Gaussian means and inverse-Wishart covariances	$\mu_1 \sim \mathsf{N}(m_1, \frac{1}{\nu_1}\Sigma_1)$, $\mu_2 \sim \mathsf{N}(m_2, \frac{1}{\nu_2}\Sigma_2)$	$\Sigma_1 \sim \mathsf{IW}(\kappa_1, \Psi_1)$,$\Sigma_2 \sim \mathsf{IW}(\kappa_2, \Psi_2)$	$m_1 = 0 \cdot \mathbf{1}_d$, $m_2 = 0.22 \cdot \mathbf{1}_d$, $\nu_1 = 120$, $\nu_2 = 20$, $\Psi_1 = \Psi_2 = 83.7 \cdot I_d$, $\kappa_1 = \kappa_2 = 300$

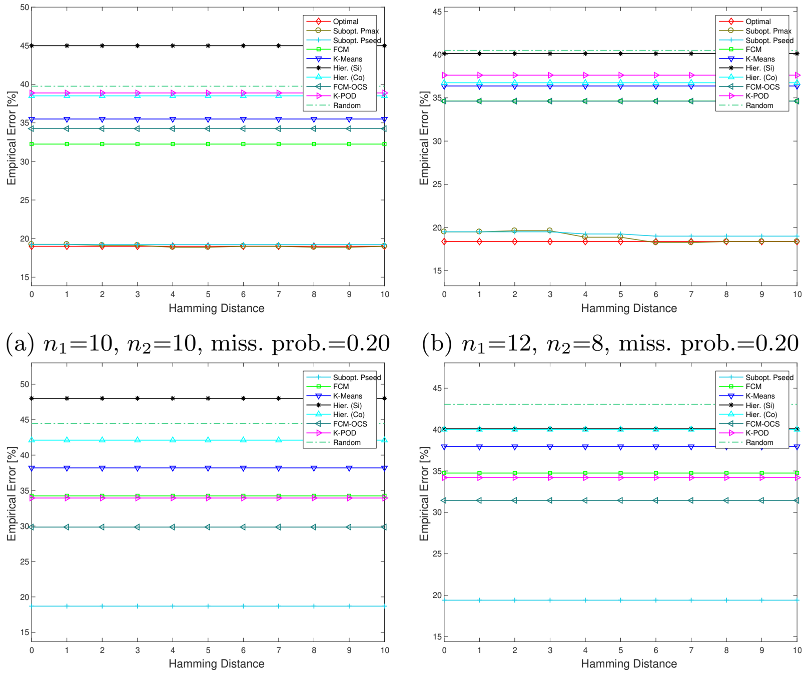

(a) n_1=10, n_2=10, miss. prob.=0.20 (b) n_1=12, n_2=8, miss. prob.=0.20

(c) n_1=25, n_2=25, miss. prob.=0.20 (d) n_1=30, n_2=20, miss. prob.=0.20

Figure S1: **Average clustering errors for the model with fixed means and fixed covariances.** The plots correspond to 20 features with missing probability of 0.2.

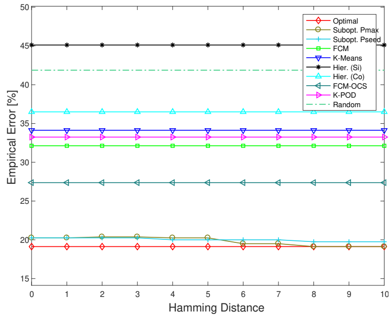

(a) n_1=10, n_2=10, miss. prob.=0.20

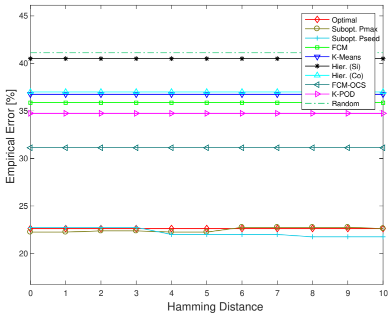

(b) n_1=12, n_2=8, miss. prob.=0.20

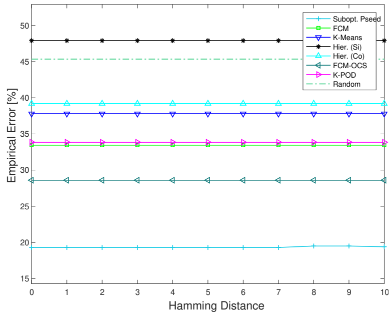

(c) n_1=25, n_2=25, miss. prob.=0.20

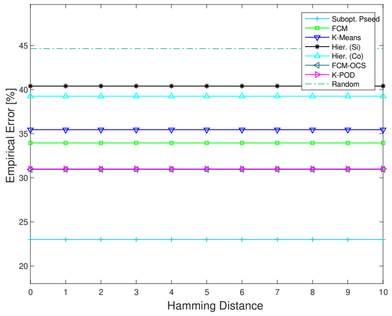

(d) n_1=30, n_2=20, miss. prob.=0.20

Figure S2: **Average clustering errors for the model with Gaussian means and fixed covariances.** The plots correspond to 20 features with missing probability of 0.2.

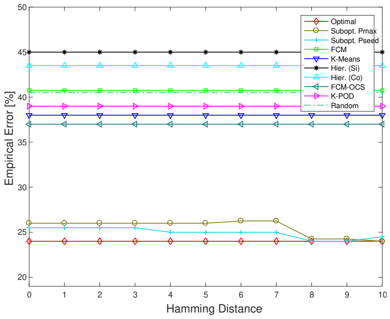

(a) n_1=10, n_2=10, miss. prob.=0.20

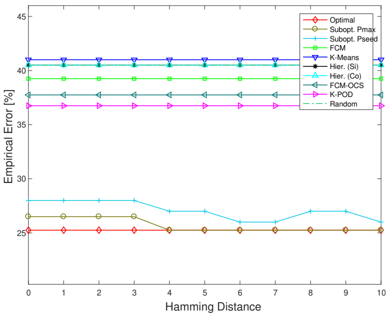

(b) n_1=12, n_2=8, miss. prob.=0.20

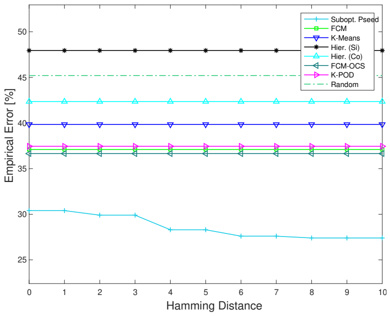

(c) n_1=25, n_2=25, miss. prob.=0.20

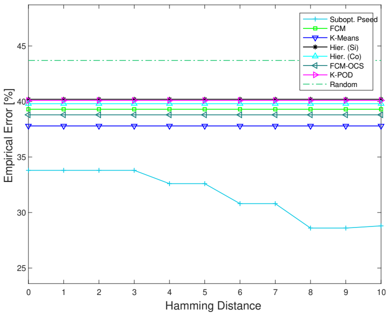

(d) n_1=30, n_2=20, miss. prob.=0.20

Figure S3: **Average clustering errors for the model with Gaussian means and inverse-Wishart covariances.** The plots correspond to 20 features with missing probability of 0.2.

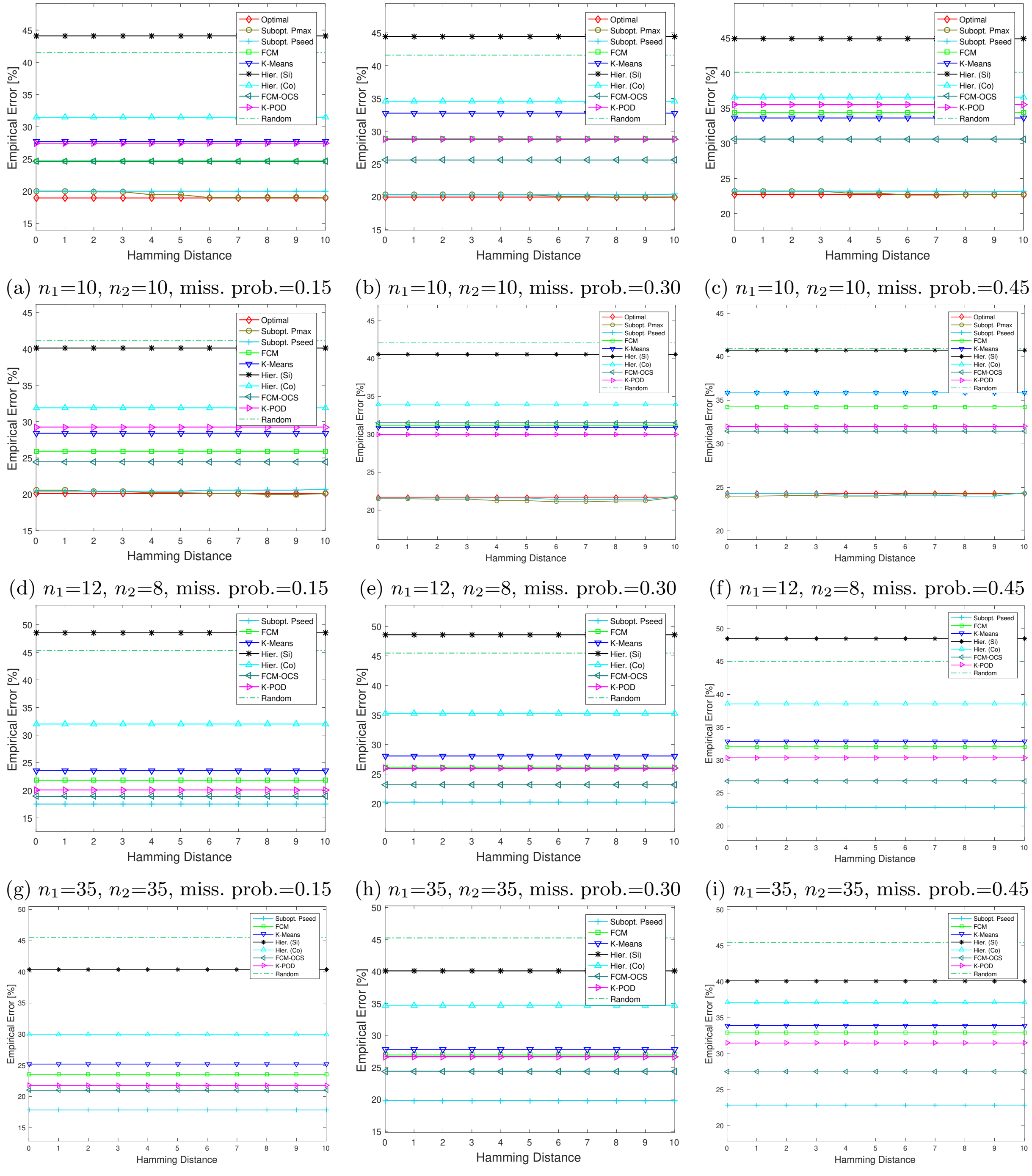

(a) n_1=10, n_2=10, miss. prob.=0.15 (b) n_1=10, n_2=10, miss. prob.=0.30 (c) n_1=10, n_2=10, miss. prob.=0.45

(d) n_1=12, n_2=8, miss. prob.=0.15 (e) n_1=12, n_2=8, miss. prob.=0.30 (f) n_1=12, n_2=8, miss. prob.=0.45

(g) n_1=35, n_2=35, miss. prob.=0.15 (h) n_1=35, n_2=35, miss. prob.=0.30 (i) n_1=35, n_2=35, miss. prob.=0.45

(j) n_1=42, n_2=28, miss. prob.=0.15 (k) n_1=42, n_2=28, miss. prob.=0.30 (l) n_1=42, n_2=28, miss. prob.=0.45

Figure S4: **Average clustering errors for the model with fixed means and covariances.** The first and second rows correspond to $n = 20$, and the third and fourth rows correspond to $n = 70$. From left to right, the columns correspond to missing probability equal to 0.15, 0.30, and 0.45, respectively.

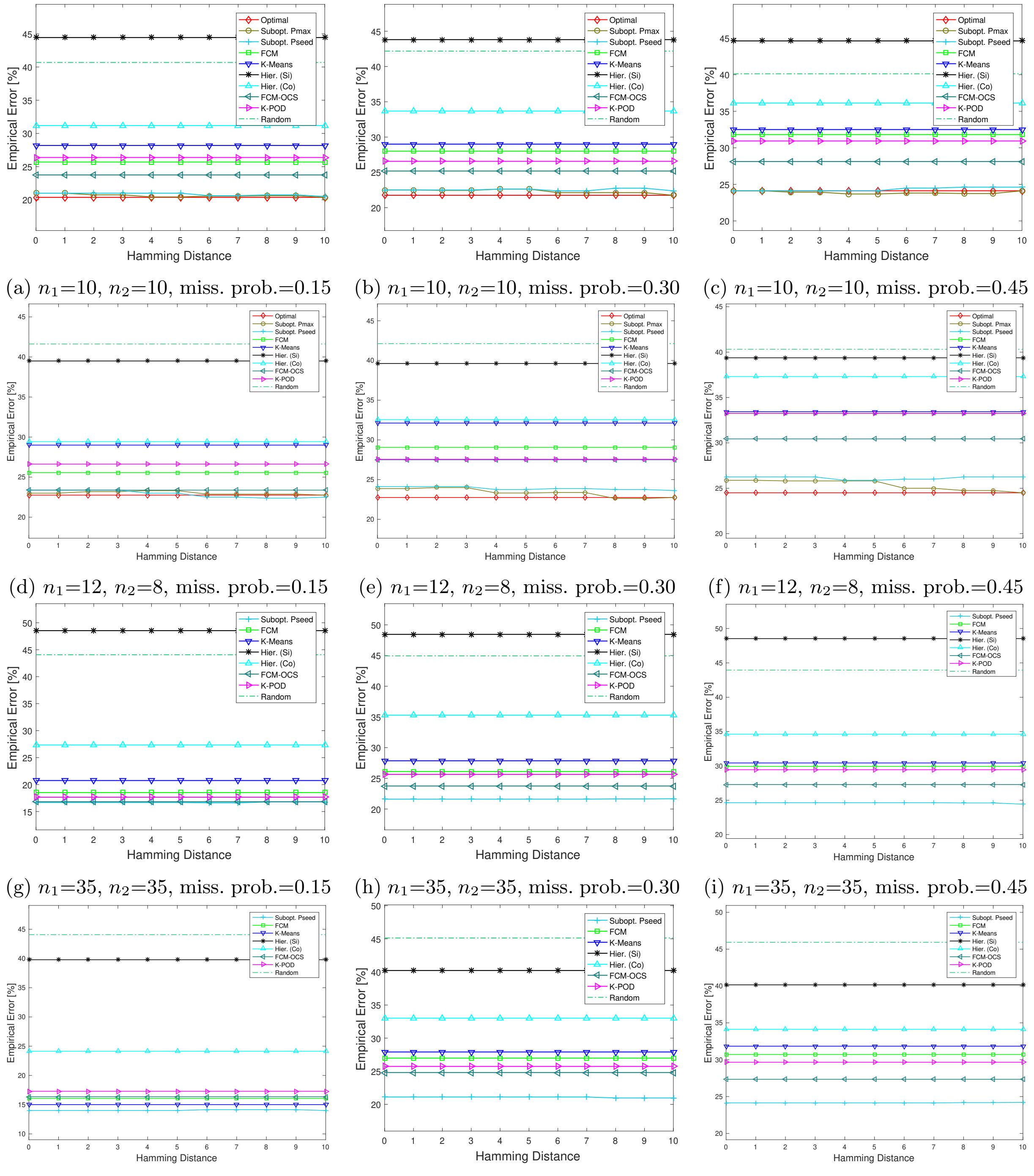

(a) n_1=10, n_2=10, miss. prob.=0.15 (b) n_1=10, n_2=10, miss. prob.=0.30 (c) n_1=10, n_2=10, miss. prob.=0.45

(d) n_1=12, n_2=8, miss. prob.=0.15 (e) n_1=12, n_2=8, miss. prob.=0.30 (f) n_1=12, n_2=8, miss. prob.=0.45

(g) n_1=35, n_2=35, miss. prob.=0.15 (h) n_1=35, n_2=35, miss. prob.=0.30 (i) n_1=35, n_2=35, miss. prob.=0.45

(j) n_1=42, n_2=28, miss. prob.=0.15 (k) n_1=42, n_2=28, miss. prob.=0.30 (l) n_1=42, n_2=28, miss. prob.=0.45

Figure S5: **Average clustering errors for the model with Gaussian means and fixed covariances.** The first and second rows correspond to n = 20, and the third and fourth rows correspond to n = 70. From left to right, the columns correspond to missing probability equal to 0.15, 0.30, and 0.45, respectively.

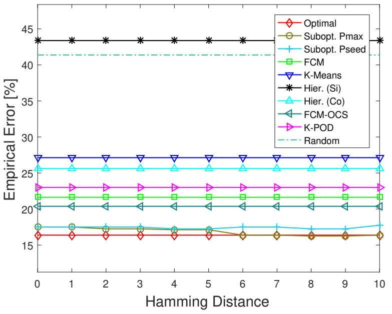

(a) n_1=10, n_2=10, miss. prob.=0.15

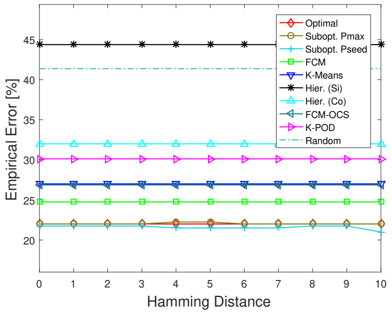

(b) n_1=10, n_2=10, miss. prob.=0.30

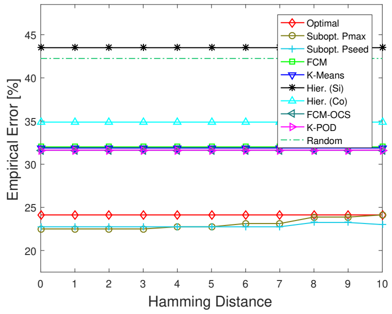

(c) n_1=10, n_2=10, miss. prob.=0.45

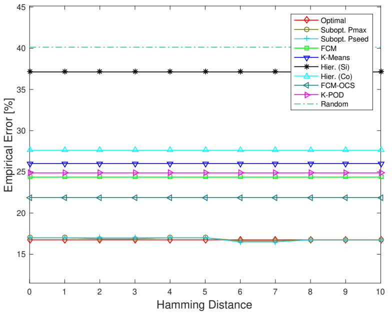

(d) n_1=12, n_2=8, miss. prob.=0.15

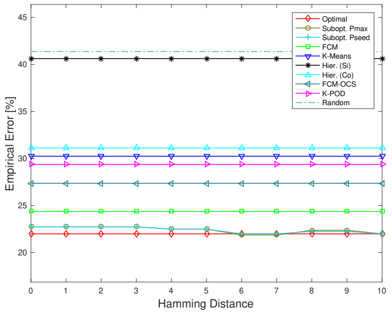

(e) n_1=12, n_2=8, miss. prob.=0.30

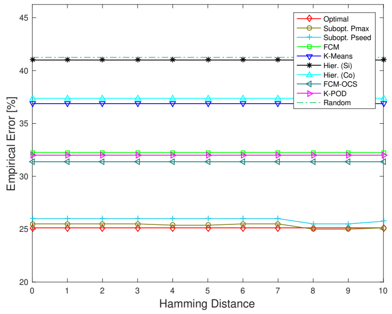

(f) n_1=12, n_2=8, miss. prob.=0.45

Figure S6: **Average clustering errors for the model with Gaussian means and inverse-Wishart covariances.** The plots correspond to $n = 20$, and from left to right, the columns correspond to missing probability equal to 0.15, 0.30, and 0.45, respectively.
